# The Mental Health of First Nations Children in Manitoba: A Population-Based Retrospective Cohort Study Using Linked Administrative Data: La santé mentale des enfants des Premières Nations au Manitoba : une étude de cohorte rétrospective dans la population, à l’aide de données administratives liées

**DOI:** 10.1177/07067437241226998

**Published:** 2024-02-11

**Authors:** Mariette J Chartier, Marni Brownell, Leona Star, Nora Murdock, Rhonda Campbell, Wanda Phillips-Beck, Mabel Horton, Chelsey Meade, Wendy Au, Jennifer Schultz, John-Michael Bowes, Brooke Cochrane

**Affiliations:** 1 Department of Community Health Sciences, Rady Faculty of Health Sciences, University of Manitoba; Winnipeg, Canada; 2 First Nations Health and Social Secretariat of Manitoba, Winnipeg, Canada; 3 525675 Manitoba First Nations Education Resource Centre, Winnipeg, Canada; 4 College of Nursing, Rady Faculty of Health Sciences, University of Manitoba, Winnipeg, Canada; 5 Advisory Working Group, First Nations Health and Social Secretariat of Manitoba, Winnipeg, Canada; 6 College of Medicine, Rady Faculty of Health Sciences, University of Manitoba. Winnipeg, Canada

**Keywords:** common mental disorders, child and adolescent psychiatry, epidemiology, suicide, cohort study, troubles mentaux communs, psychiatrie de l'enfant et de l'adolescent, épidémiologie, suicide, étude de cohorte

## Abstract

**Objective:**

First Nations children face a greater risk of experiencing mental disorders than other children from the general population because of family and societal factors, yet there is little research examining their mental health. This study compares diagnosed mental disorders and suicidal behaviours of First Nations children living on-reserve and off-reserve to all other children living in Manitoba.

**Method:**

The research team, which included First Nations and non-First Nations researchers, utilized population-based administrative data that linked de-identified individual-level records from the 2016 First Nations Research File to health and social information for children living in Manitoba. Adjusted rates and rate ratios of mental disorders and suicide behaviours were calculated using a generalized linear modelling approach to compare First Nations children (*n* = 40,574) and all other children (*n* = 197,109) and comparing First Nations children living on- and off-reserve.

**Results:**

Compared with all other children, First Nations children had a higher prevalence of schizophrenia (adjusted rate ratio (aRR): 4.42, 95% confidence interval (CI), 3.36 to 5.82), attention-deficit hyperactivity disorder (ADHD; aRR: 1.21, 95% CI, 1.09 to 1.33), substance use disorders (aRR: 5.19; 95% CI, 4.25 to 6.33), hospitalizations for suicide attempts (aRR: 6.96; 95% CI, 4.36 to 11.13) and suicide deaths (aRR: 10.63; 95% CI, 7.08 to 15.95). The prevalence of ADHD and mood/anxiety disorders was significantly higher for First Nations children living off-reserve compared with on-reserve; in contrast, hospitalization rates for suicide attempts were twice as high on-reserve than off-reserve. When the comparison cohort was restricted to only other children in low-income areas, a higher prevalence of almost all disorders remained for First Nations children.

**Conclusion:**

Large disparities were found in mental health indicators between First Nations children and other children in Manitoba, demonstrating that considerable work is required to improve the mental well-being of First Nations children. Equitable access to culturally safe services is urgently needed and these services should be self-determined, planned, and implemented by First Nations people.

## Résumé

**Objectif:**

Les enfants des Premières Nations courent un risque plus élevé de souffrir de troubles mentaux que les autres enfants de la population générale en raison de facteurs familiaux et sociétaux, et pourtant, il y a peu de recherche qui examine leur santé mentale. La présente étude compare les troubles mentaux diagnostiqués et les comportements suicidaires des enfants des Premières Nations qui vivent dans une réserve et hors réserve avec tous les autres enfants habitant au Manitoba.

**Méthode:**

L’équipe de recherche, composée de chercheurs des Premières nations et non Autochtones, a utilisé des données administratives dans la population qui liaient des dossiers individuels anonymisés du Dossier de recherche de 2016 des Premières Nations à l’information de santé et sociale pour les enfants vivant au Manitoba. Des taux ajustés et des ratios de taux des troubles mentaux et des comportements suicidaires ont été calculés à l’aide d’une approche de modélisation linéaire généralisée pour comparer les enfants des Premières Nations (n = 40 574) et tous les autres enfants (n = 197 109) et pour comparer les enfants des Premières Nations vivant dans une réserve ou hors réserve.

**Résultats:**

Comparés à tous les autres enfants, les enfants des Premières Nations avaient une prévalence plus élevée de schizophrénie [ratio de taux ajusté (RTa) : 4,42, intervalle de confiance 95 % (IC) : 3,36 à 5,82), trouble de déficit d’attention avec hyperactivité (TDAH), (RTa) :1,21, IC :1,09 à 1,33), troubles d’utilisation de substances (RTa 5,19, IC : 4,25 à 6,33), hospitalisations pour tentatives de suicide (RTa : 6,96, IC : 4,36 à 11,13) et décès par suicide (RTa : 10,63, IC : 7,08 à 15,95) ]. La prévalence du TDAH et des troubles de l’humeur/anxieux était significativement plus élevée pour les enfants des Premières Nations vivant hors réserve comparés à ceux vivant dans une réserve; par contre, les taux d’hospitalisation pour tentatives de suicide étaient deux fois plus élevés dans les réserves qu’hors réserve. Quand la cohorte de comparaison était restreinte seulement aux autres enfants des régions à faible revenu, une prévalence plus élevée de presque tous les troubles demeurait pour les enfants des Premières Nations.

**Conclusion:**

De grandes disparités ont été constatées dans les indicateurs de santé mentale entre les enfants des Premières Nations et les autres enfants du Manitoba, démontrant la nécessité d’un travail considérable afin d’améliorer le bien-être mental des enfants des Premières Nations. L’accès équitable à des services culturellement sécuritaires est nécessaire de toute urgence et ces services devraient être autodéterminés, planifiés et mis en œuvre par les peuples des Premières Nations.

Mental disorders are arguably among the most common disorders in children and adolescents and can interfere with their relationships, academic work and their prospects for the future.^1^ Recent research has found that mental illness in children and adolescents increases their risk of mental illness as adults and it also precedes other adverse outcomes such as dropping out of high school, requiring income assistance or social housing, justice system involvement and suicide deaths.^2^ Numerous risk factors are associated with mental disorders including poverty, poor nutrition, violence, and child abuse and neglect.^3^

First Nations children^a^ face a greater risk of experiencing mental disorders than other children from the general population because of historical, intergenerational and societal factors. Colonization in Canada and elsewhere has led to deplorable living conditions for many Indigenous peoples. The past centuries have seen them relegated to reserve lands, starved, neglected and forbidden from practicing their spiritual beliefs.^4,5^ Numerous Canadian laws were aimed at assimilating Indigenous peoples into Western culture and essentially preventing them from developing socially and economically.^6^ Through residential schools and the child welfare system, many children have been forcibly removed from their families, thereby threatening their language and their cultural and family ties. Many children in residential school systems endured physical, emotional and sexual abuse.^7^ These historical experiences have negatively impacted generations of children and have disrupted parenting practices. Today, First Nations people in Canada have a greater likelihood than non-First Nations people of living in overcrowded and substandard housing, experiencing food insecurity and being subjected to underfunded education and health systems. In addition, racism continues to have a profound effect on the health of children's bodies and minds through prolonged exposure to stress and through the barriers it poses to opportunities and services for Indigenous families.^8^ The lack of access to health services for First Nations people has been well documented.^9,10^ All these factors have threatened First Nations children's mental well-being. However, it is important to be aware of protective factors that can support children from these harms, including their identity, spirituality, connectedness and social support.^11^

Although the media often reports the high suicide rates among First Nations adolescents there has been surprisingly little research examining the mental health of First Nations children. For example, Lehti et al^12^ note that epidemiological data available on the mental health of Indigenous young people in the Arctic is limited and that when it comes to younger children, it is almost nonexistent. The existing research does suggest that First Nations children experience more challenges with their mental health than other children. The First Nations and Inuit Regional Health Survey found that 17% of parents thought that their child had more behaviour or emotional problems than their same-aged peers, while 25% of the children (aged 12 and over) reported having these problems.^13^ A US study of Indigenous youth aged 11 to 26 years recently reported a prevalence of psychiatric disorders of 28.7% in the past year and a cumulative lifetime prevalence of 77%.^14^ Research using 2011 Canadian Census Health and Environment Cohort (CanCHEC) data found that suicide rates among First Nations youth in Canada were 6.3 times higher than non-Indigenous youth.^15^

Understanding the prevalence of mental disorders is crucial for developing policies and providing services that address them. The Truth and Reconciliation Commission of Canada underscores the importance of reporting on mental health indicators to keep the Canadian government and the broader Canadian society accountable.^7^ The scant research on First Nations children's mental health is based on self-report survey data with only a few studies using standardized diagnostic interviews^12,14^ and existing measures have rarely been validated for First Nations children.^16^ Furthermore, there is currently a paucity of research examining schizophrenia or psychosis among Indigenous youth and very few studies report on First Nations living on- and off-reserve in a single study. It is often assumed that disproportionately higher rates of illness of First Nations rest on the high poverty experienced by them,^17,18^ and yet this has not been adequately explored.

The process by which the research is conducted is critical as well. Research that is guided by and meaningfully involves First Nations people leverages the strengths of all team members, decreases the likelihood of further stigmatization and produces mutually beneficial results.^19^ Working in strong partnerships with First Nations people also ensures that knowledge gleaned from research will benefit First Nations communities through trust building and knowledge transfer to larger networks. There is a growing literature describing ways of conducting research with Indigenous people and respecting Indigenous ways of knowing.^20–22^

Given these gaps, a research team of First Nations and non-First Nations researchers was formed to compare the percentage of diagnosed mental disorders and rates of suicidal behaviours between (a) First Nations children living on- and off-reserve and all other Manitoba children; (b) all First Nations children and only those other Manitoba children who are living in low-income areas.

## Methods

### Study Process

A partnership model with First Nations people and academic researchers was used to conduct this study. From the onset, the necessity of extensive First Nations involvement throughout all aspects of the research process was recognized. In order to respect, as closely as possible, the First Nations research principles of ownership, control, access and possession, more commonly known as OCAP, a research team was created that included both First Nations researchers and researchers who were allies. A trilateral agreement between First Nation Health and Social Secretariat of Manitoba (FNHSSM), the Manitoba government and the University of Manitoba was arranged to transfer the Government of Canada's registry of all First Nations people in Manitoba to the Manitoba Population Research Data Repository. This registry, therein called the First Nations Research File, included First Nations children living on- and off-reserve and its eligibility criteria for inclusion were laid out in the Indian Act.^23^ An advisory working group made up of Knowledge Keepers (elders from the 5 main First Nations cultural groups in Manitoba—Anishinaabe, Cree, Anishininew, Dakota and Dene peoples) and representatives from the Manitoba government was formed to guide and interpret the study and ensure effective knowledge translation. It was crucial to hear the voices of the Knowledge Keepers as they affirmed the relevance of the study, provided direction on how to present the indicators and shared valuable insights into the interpretation of the findings. This study was approved by FNHSSM's Health Information Research Governance Committee, the University of Manitoba research ethics board (No. HS21198/H2017:342), and the Health Information Privacy Committee of Manitoba Health. We did not obtain individual informed consent, given that the administrative data is de-identified and analysed within a highly secure university environment.

### Study Design and Cohort Formation

**Figure 1.**
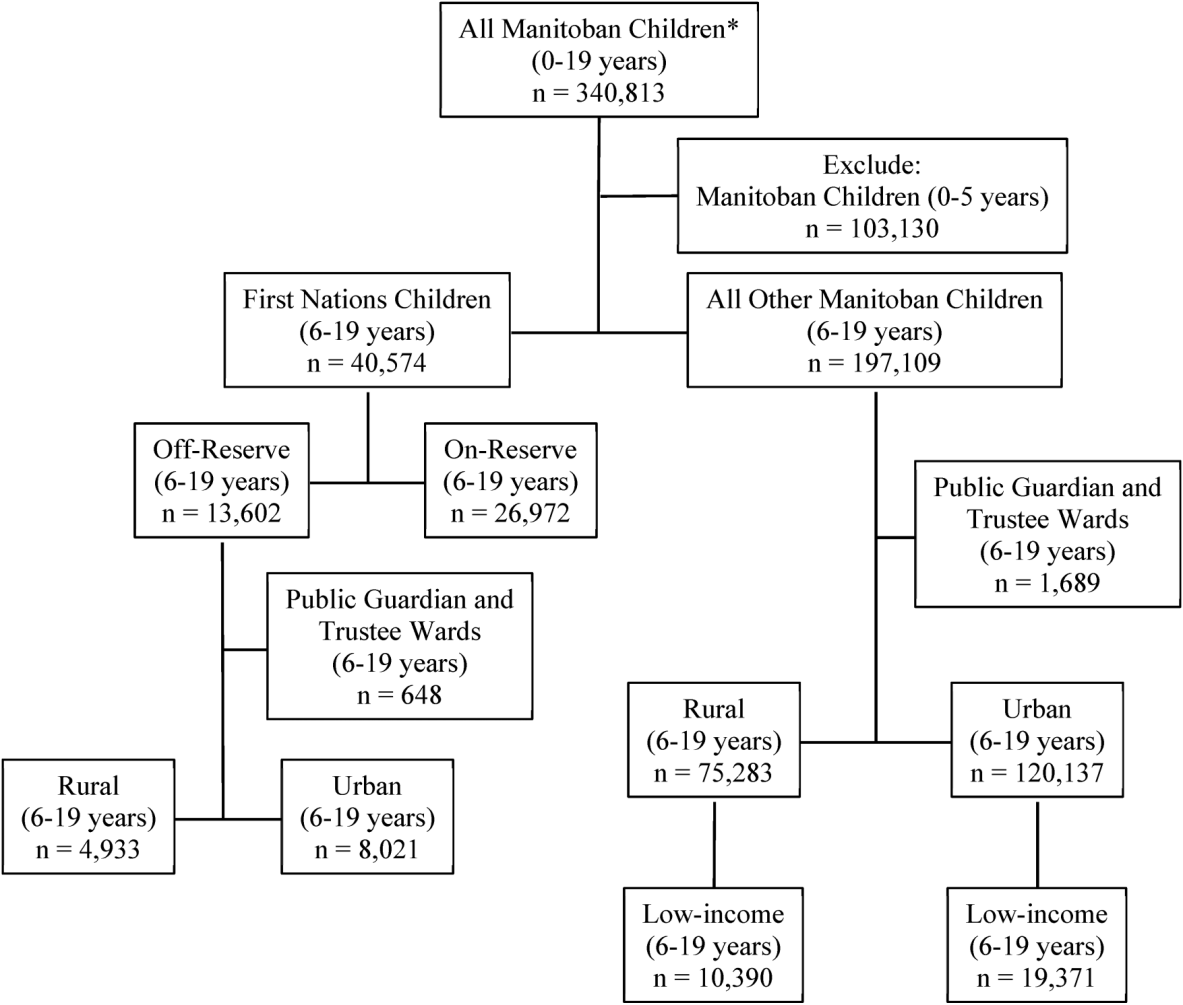
Cohort formation flowchart of First Nations children and other children, ages 6–19 years, living in Manitoba in 2016. *Children living in Manitoba in 2016 with a valid Personal Health Identification Number. Duplicates removed.

This retrospective cohort study utilized population-based administrative data that allows de-identified individual-level linkages across multiple databases through a scrambled health number. The study cohort was derived by linking the records of 340,813 children (ages 0–19 years) from the 2016 Manitoba Health Insurance Registry to the First Nations Research File. Records of children with missing health numbers, children no longer living in Manitoba or those no longer alive were excluded. We further excluded children 0–5 years old as mental health diagnosis after that age is considered more reliable. Other Manitoba children included virtually all other children living in Manitoba in 2016, including non-Indigenous children and Métis, Inuit, and non-registered First Nation children. Figure 1 illustrates that the study cohort of children (6–19 years) was made up of registered First Nations children (*n* = 40,574) and all other Manitoba children (*n* = 197,109). Among First Nations children, 13,602 (33.5%) were living off-reserve and 26,972 (66.5%) were living on-reserve at the time of the study.

To address the second research objective, we further excluded records of children under public guardians and trustee wards because we could not reliably assign these children to an income area. The cohort was further divided by rural and urban to ensure fair comparisons between the First Nations children and other Manitoba children.

### Data Sources

The databases used in this study are from the Manitoba Population Research Data Repository housed at the Manitoba Centre for Health Policy (MCHP) at the University of Manitoba. It includes data on virtually all Manitoba residents (over 99%).^24–26^ The following databases were then used to create the cohort of children and the variables: Manitoba Health Insurance Registry (age, sex, urbanicity & cohort construction); First Nations Research File (First Nations identity); physician billing claims, hospital records, and prescription data (mental disorders and suicide attempts); Vital Statistics (suicide deaths); and Canada census (area-level income).

### Diagnosed Mental Disorders and Suicide Indicators

The mental health indicators examined were mental disorders diagnosed by a physician or a nurse practitioner. The following mental disorders were for children and adolescents aged 6 to 19 years old: attention-deficit hyperactivity disorder (ADHD) and mood and anxiety disorders. Given that the prevalence of some disorders is rare for children under 13 years of age, these were examined only for adolescents aged 13 to 19 years old: substance use disorders, schizophrenia, hospitalization for suicide attempts and suicide deaths. As described in previous studies,^2^ these indicators were created using the physician claims, hospitalization records, and prescription data and are based on ICD-9-CM and ICD-10-CA diagnostic codes (see Table 1 for specific codes used). Suicides were determined through the Vital Statistics database.

**Table 1.** Case Ascertainment Criteria of Mental Health Indicators Using Administrative Data.

Indicators	Case ascertainment criteria
Attention deficit hyperactivity disorder (ADHD)	• 1 or more hospitalizations with the ICD-10-CA code: F90 in 1 fiscal year;
	• or 1 or more physician claims with the ICD-9-CM code: 314 in 1 fiscal year;
	• or 2 or more prescriptions for ADHD drugs in 1 fiscal year without the following ICD-10-CA codes in the same fiscal year: F63, F91, F92, F93, F94, G47.4;
	• or 1 prescription for ADHD drugs in 1 fiscal year with the ICD-9-CM code 314 or ICD-10-CA code F90 in the previous 3 fiscal years.
Mood and anxiety disorders	• 1 or more hospitalizations with ICD-10-CA codes: F30, F31, F32, F33, F34, F38, F40, F41, F42, F43, F53.0, F93.0;
	• or 2 or more physician visits with ICD-9-CM codes: 296, 300, 309, 311, 313.
Substance use disorder	• 1 or more hospitalizations with ICD-10-CA codes: F10–F19, F55, Z50.2, Z50.3;
	• or 1 or more physician visits with ICD-9-CM codes: 291, 292, 303, 304, 305.
Schizophrenia	• 1 or more hospitalizations with ICD-10-CA code F20;
	• or 1 or more physician visits with ICD-9-CM code 295.
Hospitalizations for attempted suicide	• 1 or more hospitalizations with ICD-10-CA code for suicide and self-inflicted injury: X60–X84; • or 1 or more hospitalizations with ICD-10-CA code for poisoning with undetermined intent: Y10–Y12, Y16, Y17;
	• or 1 or more hospitalizations with ICD-10-CA code for accidental poisoning*:* X40–X42, X44, X46, X47 combined with a psychiatric tariff code from medical claims during hospital stay or within 30 days of discharge.
Suicide deaths	• 1 Vital Statistics death record with ICD-10-CA codes for self-inflicted injury as the primary cause of death: X60–X84;
	• or 1 Vital Statistics death record with ICD-10-CA codes for poisoning with undetermined intent as the primary cause of death: Y10–Y12, Y16, Y17;
	• or 1 Vital Statistics death record with ICD-10-CA codes for accidental poisoning as the primary cause of death: X40–X42, X46, X47.

### Analytic Strategy

Adjusted rates and rate ratios were calculated using a generalized linear modelling approach (negative binomial or Poisson distribution) incorporating an interaction term. These rates were calculated over a 5-year period (2012/13 to 2016/17). Covariates included age, sex, and cohort (First Nations or all other Manitobans). Income quintiles were also included when comparing all First Nations children with other children living in low-income areas. Models were created for each mental health indicator to estimate the prevalence and rates for the indicators and to compare these across groups. All data analyses were conducted on MCHP's secure servers using SAS version 9.4 software.

## Results

Although not shown in the tables or figures, we observed that children make up a large proportion of the First Nations population. Among First Nations people, 45% are 19 years old or younger, in contrast with 25% among all other Manitobans. The age and sex distributions are similar between First Nations children and other Manitoba children and between First Nations children living on- and off-reserve.

As shown in Table 2, compared with all other Manitoba children, First Nations children had a higher prevalence of ADHD (adjusted rate ratio (aRR): 1.21, 95% confidence interval (CI), 1.09 to 1.33), substance use disorders (aRR: 5.19; 95% CI, 4.25 to 6.33), schizophrenia (aRR: 4.42; 95% CI, 3.36 to 5.82), hospitalizations for suicide attempts (aRR: 6.96; 95% CI, 4.36 to 11.13) and suicide deaths (aRR: 10.63; 95% CI, 7.08 to 15.95). However, no differences were found in mood and anxiety disorder rates between the groups of children. Rates of ADHD and mood and anxiety disorders were significantly higher for First Nations children living off-reserve compared to on-reserve, however, in contrast, hospitalization rates for suicide attempts were twice as high on-reserve than off-reserve. No differences were found in substance use disorders or schizophrenia between First Nations adolescents living on-reserve and those living off-reserve.

**Table 2.** Adjusted Prevalence of Diagnosed Mental Disorders Among First Nations and All Other Manitoba Children and Rate Ratios Comparing First Nations Children and All Other Children and Comparing First Nations Children Living On- and Off-Reserve.

	Adjusted rate (95% CI)			Adjusted rate (95% CI)		
	First Nations	AOM		FN on-reserve	FN off-reserve	
Indicator (6-19 years)	*n* = 40,574	*n* = 197,109	aRR	*n* = 26,972	*n* = 13,602	aRR
ADHD	9.25 (8.60 to 9.94)	7.66 (7.17 to 8.19)	1.21 (1.09 to 1.33)*	6.49 (5.99 to 7.03)	14.63 (13.53 to 15.83)	0.44 (0.40 to 0.50)*
Mood or anxiety	5.21 (4.55 to 5.97)	5.49 (4.83 to 6.25)	0.95 (0.79 to 1.14)	4.43 (3.84 to 5.10)	7.02 (6.07 to 8.11)	0.63 (0.52 to 0.77)*
**Indicator (13-19 years)**	***n* = 18,811**	***n* = 99,882**		***n* = 12,324**	***n* = 6,487**	
Substance use disorder	7.64 (6.63 to 8.80)	1.47 (1.28 to 1.70)	5.19 (4.25 to 6.33)*	7.73 (6.67 to 8.95)	7.49 (6.38 to 8.79)	1.03 (0.83-1.28)
Schizophrenia	0.76 (0.63 to 0.93)	0.17 (0.14 to 0.21)	4.42 (3.36 to 5.82)*	0.76 (0.61 to 0.95)	0.79 (0.60 to 1.03)	0.96 (0.69-1.35)
Suicide attempts^1^	7.30 (5.24 to 10.15)	1.05 (0.75 to 1.46)	6.96 (4.36 to 11.13)*	9.01 (6.46 to 12.59)	4.11 (2.86 to 5.90)	2.20 (1.34-3.60)*
Suicide^2^	73.86 (58.43 to 93.35)	6.95 (4.99 to 9.68)	10.63 (7.08 to 15.95)*	76.26 (57.29 to 101.49)	69.40 (46.12 to 104.43)	1.10 (0.67-1.81)

*Note*. All rates are per 100 children and adjusted by age and sex unless otherwise noted. ADHD = attention deficit hyperactviity disorder; AOM = all other Manitoban children; aRR  =  adjusted rate ratio; FN  =  First Nations.

^1^Per 1000 children. ^2^Crude rate per 100,000 children. *p < 0.01.

Tables 3 and 4 show adjusted rates and comparisons between First Nations children and other children when we restricted our cohort of other Manitoba children to only those living in low-income areas. In urban areas (Table 3), First Nations children had a higher prevalence of all mental disorders, including mood and anxiety disorders, and higher rates of suicide attempts and deaths compared to other children living in low-income areas. For example, in urban areas, First Nations adolescents were over 3 times more likely to be diagnosed with a substance use disorder than other adolescents living in low-income areas. Similarly, in rural areas (Table 4), First Nations children had a higher prevalence of almost all mental disorders, particularly substance use disorder and schizophrenia. The only exception is that no differences were found in the ADHD prevalence between First Nations children living on reserve and all other Manitoba children living in low-income rural areas.

**Table 3.** Adjusted Prevalence of Urban Children in Manitoba With Diagnosed Mental Disorders and Rate Ratios Comparing First Nations Children in Urban Areas to All Other Children Living in Low-Income Areas.

	Adjusted rate (95% CI) for urban children		
	First Nations	AOM (low income)	
Indicator (6-19 years)	n = 8,021	n = 19,371	aRR
ADHD	15.26 (13.94 to 16.69)	9.43 (8.67 to 10.26)	1.62 (1.43 to 1.83)*
Mood or anxiety	8.32 (7.20 to 9.60)	5.73 (4.98 to 6.59)	1.45 (1.19 to 1.77)*

**Indicator (13–19 years)**	***n* = 3,741**	***n* = 9,465**	
Substance use disorder	6.79 (5.67 to 8.13)	1.98 (1.63 to 2.40)	3.43 (2.64 to 4.47)*
Schizophrenia	0.85 (0.62 to 1.16)	0.31 (0.23 to 0.43)	2.71 (1.76 to 4.18)*
Suicide attempts^1^	4.15 (2.82 to 6.12)	1.14 (0.75 to 1.72)	3.66 (2.08 to 6.43)*
Suicide^2^	80.09 (48.28 to 132.85)	12.69 (5.70 to 28.24)	6.31 (2.45 to 16.27)*

*Note*. All rates are per 100 children and are adjusted by age and sex unless otherwise noted. ADHD = attention deficit hyperactviity disorder; AOM = All Other Manitoba Children; aRR = adjusted rate ratio; FN: first nations.

^1^Per 1,000 children. ^2^crude rate per 100,000 children. *p < 0.01.

**Table 4.** Adjusted Prevalence of Rural Children in Manitoba with Diagnosed Mental Disorders and Rate Ratios Comparing First Nations Children in Rural Areas to All Other Children Living in Low-Income Areas.

	Adjusted rate (95% CI)			aRR (95% CI)	
Indicator (6–19 years)	FN on-reserve *n* = 26,972	FN off-reserve *n* = 4,933	AOM (low-income) *n* = 10,390	FN on-reserve versus AOM (low-income)	FN off-reserve versus AOM (low-income)
ADHD	6.48 (5.95 to 7.05)	9.56 (8.53 to 10.72)	5.55 (4.98 to 6.17)	1.17 (1.02 to 1.34)	1.72 (1.47 to 2.02)*
Mood or anxiety	4.50 (3.94 to 5.14)	5.46 (4.63 to 6.44)	3.29 (2.80 to 3.88)	1.37 (1.11 to 1.68)*	1.66 (1.32 to 2.09)*
**Indicator (13–19 years)**	***n* = 12,308**	***n* = 2,296**	***n* = 5,237**		
Substance use disorder	7.77 (6.71 to 9.00)	8.85 (7.30 to 10.74)	2.14 (1.71 to 2.68)	3.63 (2.77 to 4.75)*	4.13 (3.07 to 5.56)*
Schizophrenia	0.75 (0.60 to 0.94)	0.63 (0.41 to 0.96)	0.14 (0.08 to 0.25)	5.28 (2.91 to 9.60)*	4.41 (2.20 to 8.86)*
Suicide attempts^1^	8.97 (6.43 to 12.53)	4.39 (2.88 to 6.70)	1.31 (0.83 to 2.07)	6.85 (3.89 to 12.06)*	3.35 (1.80 to 6.23)*
Suicide^2^	76.40 (57.40 to 101.68)	66.92 (33.47 to 133.82)	s		

*Note*. All rates are per 100 children and are adjusted by age and sex unless otherwise noted. ADHD = attention deficit hyperactviity disorder; AOM = all other Manitoba children; aRR = adjusted rate ratio; s = value suppressed due to small number; FN = First Nations. ^1^Per 1,000 children. ^2^crude rate per 100,000 children. *p < 0.01.

## Discussion

This population-based study found large disparities in mental health indicators between First Nations children and other children in Manitoba and some differences in mental health indicators between children living on- and off-reserve. Even when we restricted the cohort of other children to only those living in low-income areas, First Nations children still had a higher prevalence of almost all mental disorders and higher rates of suicide attempts and deaths. By restricting our comparison cohort in this way, we hoped to shed some light on the role of income in explaining the health disparities between First Nations children and other children. Using this restricted cohort, the disparity between groups narrowed, but remained significantly large. These results suggest that while income explains part of the disparity, other factors are clearly responsible. Racism, discrimination, colonialism, the legacy of residential schools, and the overrepresentation of First Nations children in the child welfare system are some of the factors that must also be addressed to improve the mental well-being of First Nations children.^8^

These health disparities between First Nations children and other Manitoba children are unacceptably high, particularly in a high-income country such as Canada. Given that children make up 45% of the First Nations population in Manitoba, it is imperative that their mental well-being is nurtured for the future of First Nations people. Multiple factors have been found to be associated with children's mental health thereby informing ways of addressing these disparities. Positive family cohesion, higher efficacy, higher self-esteem and optimism were associated with good mental health whereas poor parental mental health, discrimination, negative family cohesion and adverse childhood experiences were associated with poor mental health.^27–29^ There is a growing body of knowledge pointing to strategies to improve the well-being of children including initiatives that promote strong and nurturing Indigenous families,^30^ strength-based, family centred home visiting services for young Indigenous families,^31^ and school-based relationship-based mentoring programs.^32^ Knowledge Keepers on our Advisory Working Group have shared that children thrive when their communities have the resources that support children and adolescents such as recreational services, peer support groups, childcare services, land-based education and culturally safe health services. Some of this strengths-based work is already being implemented in Canada through the Inuit Tapirtt Kanatami's National Inuit Suicide Prevention Strategy.^33^

Consistent with previous research, this study found that ADHD was diagnosed at a higher rate among First Nations children compared to other Manitoba children, consistent with previous Canadian and Australian research.^34,35^ We found that this difference among the groups of children was most striking in urban settings which offers greater access to diagnostic assessment and treatments. First Nations children living on-reserve had lower rates than those living off-reserve. This may be partially explained by limited access to diagnostic and treatment services in rural areas, particularly on-reserve. Taken together, it appears that when First Nations children have access to services, they are diagnosed at higher rates than other children.

We initially found no difference in mood and anxiety disorder rates in the larger cohort between First Nations children and other children, however, when analyses were restricted to a comparison cohort of only low-income children and stratified by urban and rural, First Nations children have significantly higher rates of mood and anxiety disorders in both urban and rural areas. Similar results were found in examining the depressed mood of Indigenous Saskatchewan youth^17^ and Indigenous post-secondary students.^36^ As with ADHD, mood and anxiety disorders rates were lower among children living in rural areas and in First Nations children living on-reserve.

Our finding that First Nations adolescents were diagnosed with higher rates of substance use disorder compared to all other adolescents is consistent with previous Canadian research.^37,38^ The rate ratios were considerably attenuated when First Nations adolescents were compared to other adolescents living in low-income areas, but the gap remained statistically significant. These high substance use disorder rates may be related to poor access to mental health services, as previous studies have found that mood and anxiety disorders often precede substance use disorders.^39,40^ Other researchers suggest that enculturation or “the degree to which Aboriginal peoples identify with, feel a sense of pride for, and integrate the values and norms of their Aboriginal heritage culture” was linked to less illicit and prescription drug problems.^41^

We found higher rates of schizophrenia among First Nations adolescents compared to other Manitoba adolescents. No differences were found between First Nations adolescents living on-reserve and those living off-reserve. One comparable study was from New Zealand where Maori youth were found to be overrepresented in the schizotypal group compared to non-Aboriginal youth.^42^ A review of schizophrenia prevalence among Indigenous adults^43^ found mixed results, likely due to different methodologies used. The authors of this review noted that many studies reported a higher prevalence of schizophrenia among Indigenous compared to non-Indigenous adults, however, some studies found no differences.^43^

The disparity in rates of suicide attempts and deaths between First Nations adolescents and other adolescents found in our study is an alarming, but not a novel finding.^18,44,45^ These rates were higher among First Nations adolescents living on-reserve compared to those living off-reserve. We noted an inconsistency among First Nations children living on-reserve of relatively lower mental disorder rates, but high rates of suicide attempts and deaths. Mood and anxiety disorders appear to be underdiagnosed and likely undertreated particularly on-reserve, reflective of the wider issue of inadequate health services for First Nations people.^9,10^

This study provides a population-based perspective on the mental health of First Nations children. The Manitoba Population Research Data Repository includes data on virtually everyone in the province including First Nations children. A key strength was the partnership approach that facilitated the study. The research team included researchers from academia and coprincipal investigators from First Nations organizations. This ensured linkages between Manitoba's Data Repository and the Government of Canada's registry of First Nations people through a trilateral agreement between FNHSSM, the Government of Manitoba and the University of Manitoba and guidance was provided by Knowledge Keepers from the 5 linguistic groups in Manitoba. This approach addressed key barriers to Indigenous health data analysis, namely, the lack of high-quality First Nations identifiers^46^ and ensuring appropriate interpretation of the findings.

We acknowledge that the study findings are based on secondary data that were collected for administrative purposes in the province of Manitoba. While the diagnostic codes collected during the course of service delivery were from highly trained health providers, in many cases, the data underreport the true prevalence of illnesses. The rates of mental health indicators reported reflect children who were diagnosed by physicians and nurse practitioners. Those who were not brought to the attention of these health-care providers or those who were seen solely by other mental health providers were not counted. This is particularly a challenge in remote communities because complete data from nursing stations is lacking.

Another limitation is the lack of strength-based indicators (e.g., mental wellness, positive relationships, and social cohesion) despite the research team's strong desire to present strength-based findings. The danger with a deficit approach is that it can perpetuate colonialism and stereotyping by assuming that Indigenous people are somehow inferior. O’Keefe et al.^47^ discuss a number of socio-ecological models to guide youth mental health and wellness in research and programs, thereby ensuring that culture and context are accounted for. Innovative Indigenous scholars are providing insights into how best to produce strength-based research to guide future research.^48^ Thurber et al.^49^ propose a practical way of using administrative data in a strength-based way by simply inversing variables to report a positive outcome, for example reporting “excellent or good” mental health rather than “poor” mental health. A recent study based on perspectives from Indigenous researchers provides focus areas to define Indigenous strengths-based research including decolonizing research, intergenerational healing and flourishing, and centring Indigenous ways and cultures in research.^50^Members of our research team at FNHSSM are also at the forefront of nation-based indicators development which are positive and oriented toward measuring what is working in communities.^19,51^

As called for by the Truth and Reconciliation Commission of Canada,^7^ this study provides a clear picture of the mental health of First Nations children. The Knowledge Keepers on our Advisory Working Group remarked that the study validated what they had already observed in their communities: the stark gap between First Nations and other Manitoba children. In their words,

Our children are bearing the brunt of political decisions and inaction, from unsafe homes, unsafe drinking water, lack of health care, unresolved trauma, and the impacts of colonial policies intended to destruct rather than to build and empower. The intergenerational impacts of destructive policies and actions/inaction must stop. Our children and grandchildren have a right to a good life as was intended by our Creator.^52^

Their remarks and these findings demonstrate that a considerable amount of work is required to improve the mental well-being of First Nations children. Equitable access to culturally safe services is urgently needed and these services should be self-determined, planned, and implemented by First Nations people.

## Notes

a. In the Canadian context, the term *First Nations* refers to Indigenous individuals who are registered members of a band. *First Nations* has replaced the term *Indian* as used in the Constitution Act of 1982, which specifies that the Aboriginal Peoples of Canada consist of 3 groups: Indians (now called First Nations), Inuit, and Métis. In the province of Manitoba, there are 5 major linguistic groups among First Nations: the Anishinaabe, Cree, Anishininew, Dakota, and Dene.
